# Calculating intraocular lens power in anterior megalophthalmos: A case report

**DOI:** 10.3389/fmed.2022.926792

**Published:** 2022-08-17

**Authors:** Jiancheng Mu, Yu Yang, Tianxu Xiong, Wei Fan

**Affiliations:** Department of Ophthalmology, West China Hospital, Sichuan University, Chengdu, China

**Keywords:** anterior megalophthalmos, cataract, intraocular lens, IOL power calculation, surgical technique

## Abstract

**Introduction:**

We report a case of a man with cataract and anterior megalophthalmos (AM), in which some myopia was retained when calculating intraocular lens (IOL) power using the Haigis formula to avoid postoperative farsightedness.

**Case description:**

A 59-year-old Chinese man was referred to our clinic for cataract surgery in his right eye. He had strong bilateral megalocornea, and his left eye had undergone surgery four times. After complete preoperative examinations and repeated biometry, the Haigis formula was used, and a 3-piece IOL was implanted with a target power of −1.97 D. At 1-year follow-up, the patient showed the best-corrected distance vision of 20/20 with the refraction of −1.50 DC × 160°, and the IOL was stable.

**Conclusion:**

Our patient with anterior megalophthalmos showed postoperative hyperopia drift even though the Haigis formula was used as suggested in previous studies. To prevent farsightedness after surgery, some myopia should be retained when calculating IOL power. The Kane, Holladay II with AL adjustment, and Barrett Universal II formulas may be more accurate for calculating IOL power in such patients.

## Introduction

Anterior megalophthalmos is a rare bilateral, non-progressive congenital defect characterized by a corneal diameter greater than 13 mm, deep anterior chamber, normal intraocular pressure, and thinning cornea (1, 2). The disease is usually inherited in an X-linked manner, but other genetic patterns have also been reported (2, 3).

Individuals between 30 and 50 years who have anterior megalophthalmos may also present cataracts, glaucoma, arcus juveniles, lens subluxation, and mosaic corneal dystrophy (3, 4). Other complications have also been reported, including iris atrophy, coloboma, zonular fiber abnormalities, iris transillumination, retinal detachment, and asymmetric corneal size (1, 3, 5–7). Many cases of anterior megalophthalmos have been reported, but the best way to calculate the IOL power remains unclear. This is important to establish because choosing insufficient power may lead to postoperative hyperopia, while choosing too much power may lead to postoperative myopia. Both types of error are known as postoperative refractive error, which, even if small, can substantially affect visual outcomes (8). Unlike other corneal abnormalities, such as keratoglobus or keratoconus (9), the regularity and refractive power of the cornea are usually normal in eyes with anterior megalophthalmos, in which the main factors that affect the accuracy of IOL power calculation are the abnormal anterior chamber depth (ACD) and the difficulty in predicting the effective lens position (ELP). The error in ELP prediction and the inappropriate formula selection are the main causes of postoperative refractive error. In addition, the influence of wide and relaxed capsular bags on the stability of IOL is another major factor affecting the outcome of surgery in patients with anterior megalophthalmos.

Here we describe how we calculated IOL power for a patient with anterior megalophthalmos by retaining some myopia and thereby reducing farsightedness drift after implanting a three-piece IOL. Nevertheless, the patient still showed some hyperopia drift, leading us to compare the calculation outcomes from various formulas and determine that the Kane, Holladay II with AL adjustment, and Barrett Universal II formulas may be more accurate in patients with anterior megalophthalmos.

## Case presentation

**FIGURE 1:**
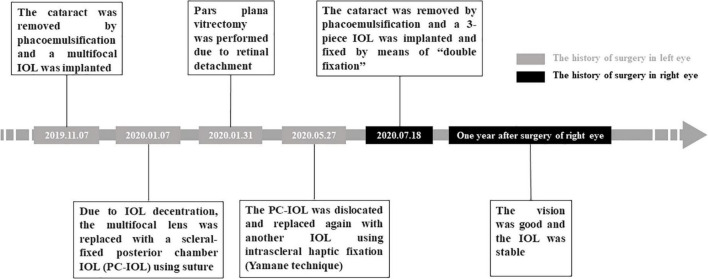
Timeline of the major surgical interventions in the patient’s both eyes.

A 59-year-old Chinese man was admitted to the ophthalmology clinic of West China Hospital of Sichuan University on 13 July 2020 due to a gradual loss of visual acuity in the right eye over the previous 5 years. His left eye had undergone numerous surgical interventions due to postoperative complications, including IOL decentration, IOL dislocations, and retinal detachment (see Figure 1). Other than that, the patient reported no history of hypertension, diabetes, trauma, or familial-hereditary diseases.

Upon admission to our hospital, the best corrected visual acuity (BCVA) in the right eye only allowed counting fingers. The BCVA of the left eye was 20/25, and refraction was +3 DS/-5 DC × 165°. Horizontal corneal diameters were enlarged in both eyes and exceeded 13 mm as measured using the IOLMaster700 (Zeiss, Jena, Germany). The main biometric measures are summarized in Table 1.

**TABLE 1:** Preoperative measurements of the patient.

Measurement	Right eye	Left eye (Pseudophakic)
IOP	12.8 mmHg	17.9 mmHg
AL	25.98 mm	26.40 mm
ACD	5.59 mm	5.94 mm
LT	3.76 mm	0.53 mm
CCT	497 μm	491 μm
WTW	13.1 mm	13.3 mm
K1/K2	43.70 D × 159°/45.55 D × 69°	41.58 D × 164°/47.56 D × 74°
Count of corneal endothelial cells	2901.6/mm^2^	1466.7/m^2^

IOP, intraocular pressure; AL, axial length; ACD, anterior chamber depth; LT, lens thickness; CCT, central corneal thickness; WTW, white-to-white distance.

**FIGURE 2:**
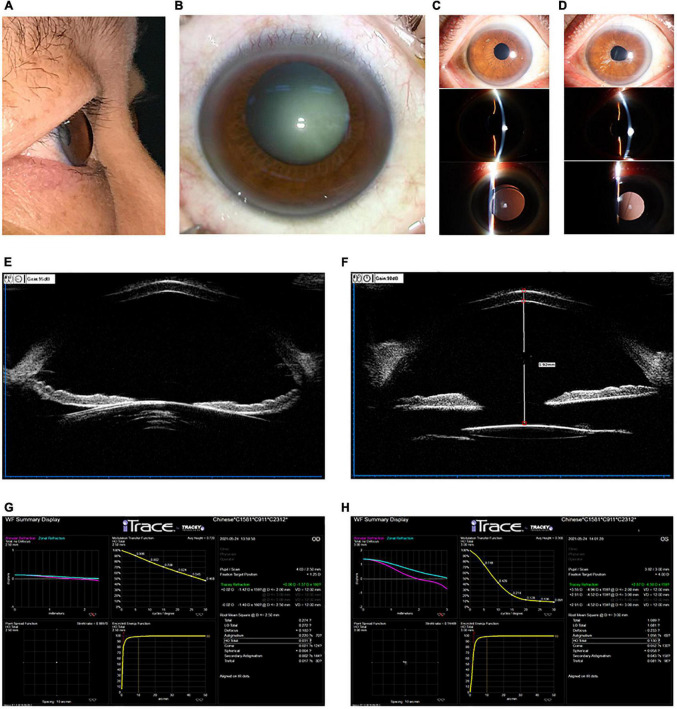
**(A,B)** Color photography of the right eye before surgery showed cataract and a significantly deepened anterior chamber. **(C)** Photographs of the right eyes by slit-lamp microscopy at 1 year after surgery at our hospital. The IOL was stably positioned in the eye. **(D)** Photographs of the left eyes by slit-lamp microscopy at 1 year after the right eye surgery at our hospital. The left iris pigment was dispersed. **(E,F)** Ultrasound biomicrographs of the right eye before **(E)** and after **(F)** surgery at our hospital. Panel **(E)** showed a deep anterior chamber and elongated zonular fibers. The panel **(F)** revealed the IOL to be centrally located in the eye after surgery. **(G,H)** Aberrometry using the iTrace system, showing visual quality in the right eye **(G)** and left eye **(H)** after right eye surgery at our hospital.

Slit lamp examination revealed unremarkable conjunctiva, an enlarged and clear cornea, a deepened anterior chamber in both eyes (Figure 2A), normal iris in the right eye but dispersion of iris pigment in the left eye, and an opaque lens in the right eye (Figure 2B). The artificial lens in the left eye was slightly off-center. Ultrasound biomicroscopy showed that the anterior chamber of the right eye was deep, and the zonular fiber of the right eye was stretched (Figure 2E). Optical coherence tomography (OCT) revealed no obvious abnormality in the fundus of either eye. The patient was diagnosed with a cataract in the right eye and anterior megalophthalmos in both eyes.

Given the unusual anatomy of our patient’s right eye, we decided to calculate IOL power using the Haigis formula, which has been reported as accurate regardless of axial length (10) and has been recommended for patients with anterior megalophthalmos (11). During the calculation of IOL power, we retained myopia to prevent hyperopic drift.

On 18 July 2020 (Figure 1), an experienced surgeon (F.W.) performed a temporal clear corneal incision of 2.75 mm long with the patient under topical anesthesia, then instilled a viscoelastic agent (DisCoVisc, Alcon) to maintain normal anterior chamber depth. The surgeon performed continuous curvilinear capsulorhexis, hydrodissecti on and hydrodelineation, followed by phacoemulsification (Stellaris, Bausch and Lomb, Rochester, NY, United States). A 3-piece hydrophobic acrylic foldable IOL (+14.5 D, targeting −1.97 D; Sensar AR40, AMO, Santa Ana, CA, United States) was implanted into the capsular bag with the technique of “double fixation,” i.e., the haptics in the capsular bag but the optical part of the IOL captured in front of the anterior capsule opening, which could make IOL more stable in the eye with a large and loose bag. The residual viscoelastic agent was aspirated, then the incision was hydrated with a balanced salt solution and checked for water tightness. During surgery, the height of the infusion bottle was lowered in light of the eye’s zonular weakness and deep anterior chamber. After surgery, the patient was given eye drops containing tobramycin and dexamethasone and eye drops containing 0.1% sodium diclofenac for 1 month.

At 1 year after surgery (Figure 1), the uncorrected visual acuity was 20/25 in the right eye and 20/80 in the left eye, while intraocular pressure was 11.3 mmHg in the right eye and 14.6 mmHg in the left. Refraction was −1.50 DC × 160° in the right eye and +1.50 DS/−2.50 DC × 170° in the left. Both eyes showed no obvious signs of inflammatory reaction and appeared to be stable, with best-corrected distance vision of 20/20.

In the right eye, the IOL was properly positioned and stable (Figure 2C), although the anterior chamber was 5.92 mm deep based on ultrasound biomicroscopy (Figure 2F). Using the iTrace system (Tracey Technologies, Houston, TX, United States), berrometry indicated good visual quality in the right eye: the average height from the modulation transfer function curve was 0.720, and the Strehl ratio from the point spread function was 0.88970 (Figure 2G). The left eye appeared stable (Figure 2D), though it gave worse visual quality than the right eye based on aberrometry (Figure 2H). Overall, cataract surgery maintained an adequate vision for daily living, and the patient felt satisfied.

Given that our patient still showed some farsightedness drift despite our retaining some myopia during the calculation of the IOL power, we compared various formulas for their ability to estimate IOL power. Our goal was to identify the most accurate formula that would lead to the smallest postoperative refractive error for the challenging case of anterior megalophthalmos. We found that the Kane, Holladay II with AL adjustment, and Barrett Universal II formulas gave the minimal postoperative refraction error (Table 2).

**TABLE 2:** Comparison of calculation outcomes from different formulas in the case of a Sensar AR40 lens (+14.5 D).

Parameter	Formula					
	Haigis	SRK/T	Hill-RBF^a^	Barrett Universal II	Holladay II with AL adjustment	Kane^b^
Predicted refraction (D)	−1.97	−1.64	−1.47	−1.42	−1.19	−1.05
Actual postoperative spherical equivalent (D)	−0.75	−0.75	−0.75	−0.75	−0.75	−0.75
Error (D)	+1.22	+0.89	+0.72	+0.67	+0.44	+0.30
IOL power (target diopter 0 D)	+11.5	+12.0	+12.0	+12.5	+12.5	+13.0

Spherical Equivalent (SE) = spherical diopter + 1/2 cylindrical diopter. Our patient had an SE of −0.75 D at a 1-year follow-up. Error = Actual Postoperative SE − Predicted Refraction.

^a^ACD used in the Hill-RBF formula was 5.25 mm (maximum). ^b^ACD used in the Kane formula was 5 mm (maximum).

## Discussion

The diagnosis of anterior megalophthalmos is challenging and requires differentiation from primary megalocornea, global cornea, and congenital glaucoma (5, 12). The diagnosis in the present patient was fairly straightforward based on bilateral cornea diameter expansion to more than 13 mm, a deep anterior chamber, an expanded ciliary ring, stretched and lose zonular fibers, and presence of cataract, yet normal intraocular pressure and relatively young age.

However, determining the appropriate IOL diopter is a challenge, particularly in the presence of anatomical abnormalities, which are characteristic of anterior megalophthalmos, such as the large cornea, deep anterior chamber, and big capsular bag, which make the effective position of IOL hard to be correctly predicted. The error in the calculation of IOL power will lead to postoperative refractive error, blurred uncorrected visual acuity, poor visual quality, and so on, seriously affecting the quality of life and satisfaction of patients. Severe cases can lead to anisometropia and even IOL replacement surgery. In addition to accurate preoperative biometrics, it is particularly important to select the appropriate calculation formula to get the right IOL power. Studies have suggested that the Holladay II formula may be superior to the SRK/T formula for predicting IOL diopter, but neither seems ideal and the two may lead to similar clinical outcomes (13, 14). Other work has suggested that the Haigis formula may be superior to those other two formulas (11), but our analysis suggests that even that formula can lead to postoperative farsightedness drift (Table 2).

Therefore, some myopic refraction should be retained to avoid residual hyperopia (13). For our patient, we applied the Haigis formula and set the target diopter to −1.97 D to prevent hyperopic drift. Even so, the operated eye did show some 1.22 D-farsightedness drift. Postoperative analysis of outcomes from multiple formulas suggested that the Kane, Holladay II with AL adjustment, and Barrett Universal II formulas might have been more suitable for our patient (Table 2). We, therefore, recommend using these formulas to calculate IOL power for patients with anterior megalophthalmos, while retaining some myopia (−0.5 to −1 D) to prevent postoperative farsightedness drift.

Another challenge for patients who have anterior megalophthalmos is removing their cataracts because of the large ciliary rings and weak zonular fibers (15). Iris atrophy in such patients often makes it difficult to dilate the pupil (1), which increases the difficulty of capsulorhexis and IOL implantation. In addition, zonular fiber hypoplasia and weakness increase the risk of IOL dislocation after surgery (16). Ultrasound biomicroscopy may be particularly helpful for determining bag size in complicated cases, such as anterior megalophthalmos (16).

The enlarged anterior segment in patients with anterior megalophthalmos can result in IOL decentering after surgery. We implanted in our patient a 3-piece IOL, which can be effective against such decentering (13), as can iris suturing, scleral fixation, or an iris-claw IOL (16, 17). In addition to choosing the right type of IOL, some special surgical designs could also bring more benefits to unusual patients, for instance, a precautionary stabilizing suture across the RK incision adjacent to the main tunnel or a double-safe suture technique may have a lower risk of dehiscence during phacoemulsification in patients with cataract with previous RK (18, 19). In our case, we implanted IOL using a “double fixation” technique, where both haptics of the IOL was positioned in the bag, and the optics were captured in the anterior capsular opening. By 1-year follow-up, the IOL in our patient was well-centered and showed relatively long-term stability. This suggests that when the capsule bag is large and loose, a “double fixation” of the IOL may be preferable.

The complicated history of the left eye of our patient, who suffered cataract, lens dislocation, and retinal detachment, highlights the need to monitor the contralateral eye for adverse developments. In the operated eye, the location of the IOL and the condition of the fundus should be monitored carefully during follow-up.

## Conclusion

Here, we describe a challenging case of anterior megalophthalmos, its surgical management, and follow-up examinations. Our patient showed relatively good visual outcomes with a stable IOL in position 1-year postoperatively. Although this is a case report and our findings should be replicated in a larger sample, the experience of diagnosis and treatment of this case gives a lot of inspiration and thinking to clinical practice. Our patient with anterior megalophthalmos showed postoperative hyperopia drift even though the Haigis formula was used, as reported in previous literature. The comparison of different IOL power calculation formulas showed that the Kane, Holladay II with AL adjustment, and Barrett Universal II formulas may be more appropriate for calculating IOL power in such patients. Some myopia may be retained when calculating IOL power for these patients to prevent farsightedness after surgery. Besides, the surgical technique of “double fixation” is a practical approach to stabilizing IOL in patients with anterior megalophthalmos.

## Data availability statement

The original contributions presented in the study are included in the article/supplementary material, further inquiries can be directed to the corresponding author.

## Ethics statement

Written informed consent was obtained from the individual(s) for the publication of any potentially identifiable images or data included in this article.

## Author contributions

JM drafted the manuscript, collected information from the patients, and interpreted the patient data. TX and YY collected information from the patients. WF made a critical revision of the manuscript and contributed to supervision and final approval. All authors read and approved the final manuscript.

## References

[1] Meire FM. Megalocornea. Clinical and genetic aspects. *Doc Ophthalmol.* (1994) 87:1–121. 10.1007/BF01676641 7835180

[2] Webb TR Matarin M Gardner JC Kelberman D Hassan H Ang W X-linked megalocornea caused by mutations in CHRDL1 identifies an essential role for ventroptin in anterior segment development. *Am J Hum Genet.* (2012) 90:247–59. 10.1016/j.ajhg.2011.12.019 22284829 PMC3276677

[3] Chlasta-Twardzik E Nowinska A Was P Jakubowska A Wylegala E. Traumatic cataract in patient with anterior megalophthalmos: case report. *Medicine (Baltimore).* (2017) 96:e7160. 10.1097/MD.0000000000007160 28746174 PMC5627800

[4] Saatci AO Soylev M Kavukcu S Durak I Saatci I Memisoglu B. Bilateral megalocornea with unilateral lens subluxation. *Ophthalmic Genet.* (1997) 18:35–8. 10.3109/13816819709057881 9134548

[5] Ahmadieh H Banaee T Javadi MA Jafarinasab MR Yazdani S Sajjadi H. Vitreoretinal disorders in anterior megalophthalmos. *Jpn J Ophthalmol.* (2006) 50:515–23. 10.1007/s10384-006-0370-9 17180525

[6] Guixeres Esteve MC Pardo Saiz AO Martinez-Costa L Gonzalez-Ocampo Dorta S Sanz Solana P. Surgical management of a patient with anterior megalophthalmos, lens subluxation, and a high risk of retinal detachment. *Case Rep Ophthalmol.* (2017) 8:61–6. 10.1159/000456068 28203198 PMC5301089

[7] Kuchenbecker J Behrens-Baumann W. Ciliary body dysplasia in megalophthalmos anterior diagnosed using ultrasound biomicroscopy. *Eye (Lond).* (2002) 16:638–9. 10.1038/sj.eye.6700156 12194082

[8] Filip M Apostol S. [Post-operatory biometry and refraction results estimated and refraction surprises–clinical study]. *Oftalmologia.* (2003) 56:11–4. 12886674

[9] Krysik K Lyssek-Boron A Janiszewska-Bil D Wylegala E Dobrowolski D. Impact of ultrasound and optical biometry on refractive outcomes of cataract surgery after penetrating keratoplasty in keratoconus. *Int J Ophthalmol.* (2019) 12:949–53. 10.18240/ijo.2019.06.11 31236351 PMC6580200

[10] Hoffer KJ Savini G. IOL Power Calculation in Short and Long Eyes. *Asia Pac J Ophthalmol (Phila).* (2017) 6:330–1. 10.22608/APO.2017338 28780778

[11] Miao A Zhang K Yu J He W Lu Y Zhu X. How many challenges we may encounter in anterior megalophthalmos with white cataract: a case report. *BMC Ophthalmol.* (2019) 19:122. 10.1186/s12886-019-1133-y 31146719 PMC6543662

[12] Gupta N Ganger A. Keratoglobus: a close entity to megalophthalmos. *Springerplus.* (2016) 5:634. 10.1186/s40064-016-2307-1 27330900 PMC4870505

[13] Assia EI Segev F Michaeli A. Cataract surgery in megalocornea Comparison of 2 surgical approaches in a single patient. *J Cataract Refract Surg.* (2009) 35:2042–6. 10.1016/j.jcrs.2009.06.043 19969205

[14] De la Parra-Colin P Barrientos-Gutierrez T Mian SI. Axial length’s role in intraocular lens power calculation error in X-linked megalocornea: a case-series analysis. *Ophthalmic Genet.* (2014) 35:180–3. 10.3109/13816810.2013.804099 24001017

[15] Sharan S Billson FA. Anterior megalophthalmos in a family with 3 female siblings. *J Cataract Refract Surg.* (2005) 31:1433–6. 10.1016/j.jcrs.2004.11.057 16105619

[16] Galvis V Tello A Rangel CM Carreno NI Berrospi RD Nino CA. Iris-clip versus iris-claw intraocular lenses. *J Cataract Refract Surg.* (2018) 44:1407. 10.1016/j.jcrs.2018.08.007 30368364

[17] Sati A Murthy SI Arjun S Rathi VM. Anterior megalophthalmos: is visual restoration possible? *Oman J Ophthalmol.* (2018) 11:184–6. 10.4103/ojo.OJO_165_2015 29930460 PMC5991068

[18] Meduri A Oliverio G Severo AA Camellin U Rechichi M Aragona P. Double safe suture during cataract surgery on post radial keratotomy patients using clear corneal incisions. *Eur J Ophthalmol.* (2022) 32:1828–32. 10.1177/11206721221083799 35229692

[19] Meduri A Urso M Signorino GA Rechichi M Mazzotta C Kaufman S. Cataract surgery on post radial keratotomy patients. *Int J Ophthalmol.* (2017) 10:1168–70. 10.18240/ijo.2017.07.23 28730124 PMC5514283

